# Supplementary material for: Discrete viral E2 lysine residues and scavenger receptor MARCO are required for clearance of circulating alphaviruses
Source: eLife. 2019 Oct 9;8:e49163. doi: 10.7554/eLife.49163 (PMC6839921; doi:10.7554/eLife.49163)
Supplement: Supplementary file 1. [file elife-49163-supp1.docx]

**Supplementary File 1.** Primers used to generate mutant viruses through site-directed mutagenesis.

| **Virus** | **Primer Forward (5′-3′)** | **Primer Reverse (5′-3′)** |
| --- | --- | --- |
| AF15561  E2 K200A | CAGACGGTGCGGTACGCGTGTAATTGCGGTGACTC | GAGTCACCGCAATTACACGCGTACCGCACCGTCTG |
| AF15561  E2 K200D | GTCAGACGGTGCGGTACGATTGTAATTGCGGTGACTC | GAGTCACCGCAATTACAATCGTACCGCACCGTCTGAC |
| AF15561  E2 K200H | GTCAGACGGTGCGGTACCACTGTAATTGCGGTGACTC | GAGTCACCGCAATTACAGTGGTACCGCACCGTCTGAC |
| AF15561  E2 K200L | ATAGTCAGACGGTGCGGTACCTGTGTAATTGCGGTGACTCAAA | TTTGAGTCACCGCAATTACACAGGTACCGCACCGTCTGACTAT |
| AF15561  E2 K200Q | CAGACGGTGCGGTACCAGTGTAATTGCGGTG | CACCGCAATTACACTGGTACCGCACCGTCTG |
| AF15561  E2 K200S | ATAGTCAGACGGTGCGGTACAGCTGTAATTGCGGTGAC | GTCACCGCAATTACAGCTGTACCGCACCGTCTGACTAT |
| ONNV SG650 E2 K200R | GCCAGACAGTACGATACAGGTGCAAATGTGACGGC | GCCGTCACATTTGCACCTGTATCGTACTGTCTGGC |
| 99659  E2 K200R | CAGACGGTGCGGTACAGGTGCAATTGTGGTGACTC | GAGTCACCACAATTGCACCTGTACCGCACCGTCTG |
| SL15649  E2 K200R | CCACCGCAATTACACCTGTACCGCACCGTCT | AGACGGTGCGGTACAGGTGTAATTGCGGTGG |
| 37997  E2 K200R | GACGGTGCGGTACAGGTGCAACTGCGGTG | CACCGCAGTTGCACCTGTACCGCACCGTC |
| RRV DC5692  E2 K251R | GCTGATCAGACAGCTAGGAGGGGCAAAGTGCAT | ATGCACTTTGCCCCTCCTAGCTGTCTGATCAGC |
| AF15561  E2 K252R | gctgaactcggggaccgaagaggaaaagttcac | gtgaacttttcctcttcggtccccgagttcagc |
| AF15561  E2 N263Q | tccgtttcctctggcacaggtgacatgcagggtgc | gcaccctgcatgtcacctgtgccagaggaaacgga |
| AF15561  E2 N273Q | gtgcctaaggcaagg**c**a**g**cccaccgtgacgtac | gtacgtcacggtgggctgccttgccttaggcac |
| RRV T48  E2 R251A | GCTGATCAGACAGCTAGGGCGGGCAAAGTGCATGTTCC | GGAACATGCACTTTGCCCGCCCTAGCTGTCTGATCAGC |
| RRV T48  E2 R251D | GCTGATCAGACAGCTAGGGATGGCAAAGTGCATGTTCC | GGAACATGCACTTTGCCATCCCTAGCTGTCTGATCAGC |
| RRV T48  E2 R251Q | GCTGATCAGACAGCTAGGCAGGGCAAAGTGCATGTTCC | GGAACATGCACTTTGCCCTGCCTAGCTGTCTGATCAGC |
| RRV T48  E2 R251S | GCTGATCAGACAGCTAGGAGCGGCAAAGTGCATGTTCC | GGAACATGCACTTTGCCGCTCCTAGCTGTCTGATCAGC |
