# Supplementary material for: Discrete viral E2 lysine residues and scavenger receptor MARCO are required for clearance of circulating alphaviruses
Source: eLife. 2019 Oct 9;8:e49163. doi: 10.7554/eLife.49163 (PMC6839921; doi:10.7554/eLife.49163)
Supplement: Supplementary file 2. [file elife-49163-supp2.docx]

| **Key Resources Table** | | | | |
| --- | --- | --- | --- | --- |
| **Reagent type (species) or resource** | **Designation** | **Source or reference** | **Identifiers** | **Additional information** |
| Cell line (*Cercopithecus aethiops)* | Vero | ATCC CCL81 | RRID:CVCL_0059 |  |
| Cell line (*Mesocricetus auratus)* | BHK-21 | ATCC CCL-10 | RRID:CVCL_1915 |  |
| Cell line (*Homo sapien*) | Human dermal fibroblasts | ATCC PCS-201-012 |  |  |
| Cell line (*Aedes albopictus)* | C6/36 | ATCC CRL-1660 | RRID:CVCL_Z230 | Cultured at 30°C |
| recombinant DNA reagent | pAF15561 (Infectious cDNA clone of Asian strain of CHIKV) | PMID:25142598 |  | Used to derive infectious virus through electroporation of RNA into cells. |
| recombinant DNA reagent (Infectious cDNA clone of CHIKV) | pAF15561 E2 K200R | PMID:28747508 |  | Used to derive infectious virus through electroporation of RNA into cells. |
| recombinant DNA reagent (Infectious cDNA clone of CHIKV) | pAF15561 E2 K200A | This paper. |  | Derived through sight directed mutagenesis (SDM) of pAF15561. Used to make infectious virus through electroporation of RNA into cells |
| recombinant DNA reagent (Infectious cDNA clone of CHIKV) | pAF15561 E2 K200D | This paper. |  | Derived through SDM of pAF15561. Used to make infectious virus through electroporation of RNA into cells. |
| recombinant DNA reagent (Infectious cDNA clone of CHIKV) | pAF15561 E2 K200L | This paper. |  | Derived through SDM of pAF15561. Used to make infectious virus through electroporation of RNA into cells. |
| recombinant DNA reagent (Infectious cDNA clone of CHIKV) | pAF15561 E2 K200Q | This paper. |  | Derived through SDM of pAF15561. Used to make infectious virus through electroporation of RNA into cells. |
| recombinant DNA reagent (Infectious cDNA clone of CHIKV) | pAF15561 E2 K200S | This paper. |  | Derived through SDM of pAF15561. Used to make infectious virus through electroporation of RNA into cells. |
| recombinant DNA reagent (Infectious cDNA clone of CHIKV) | pAF15561 E2 K200H | This paper. |  | Derived through SDM of pAF15561. Used to make infectious virus through electroporation of RNA into cells. |
| recombinant DNA reagent | p99659 (Infectious cDNA clone of Asian-American strain of CHIKV) | PMID:29138302 |  | Used to derive infectious virus through electroporation of RNA into cells. |
| recombinant DNA reagent | p99659 E2 K200R | This paper. |  | Derived through SDM of p99659. Used to make infectious virus through electroporation of RNA into cells. |
| recombinant DNA reagent | pSL15649  (Infectious cDNA clone of ECSA strain of CHIKV) | PMID:21224040 |  | Used to derive infectious virus through electroporation of RNA into cells. |
| recombinant DNA reagent | pSL15649  E2 K200R | This paper. |  | Derived through SDM of pSL15649. Used to make infectious virus through electroporation of RNA into cells. |
| recombinant DNA reagent | p37997 (Infectious cDNA clone of WA strain of CHIKV) | PMID:16102421 |  | Used to derive infectious virus through electroporation of RNA into cells. |
| recombinant DNA reagent | p37997 E2 K200R | This paper. |  | Derived through SDM of p37997. Used to make infectious virus through electroporation of RNA into cells. |
| recombinant DNA reagent | pRR64 (Infectious cDNA clone of RRV strain T48) | (Doherty et al., 1963); PMID:1673812 |  | Used to derive infectious virus through electroporation of RNA into cells. |
| recombinant DNA reagent | pRR64 E2 R251K | PMID:23514884 |  | Used to derive infectious virus through electroporation of RNA into cells. |
| recombinant DNA reagent | pRR64 E2 R251D | This paper. |  | Derived through SDM of pRR64. Used to derive infectious virus through electroporation of RNA into cells. |
| recombinant DNA reagent | pRR64 E2 R251A | This paper. |  | Derived through SDM of pRR64. Used to derive infectious virus through electroporation of RNA into cells. |
| recombinant DNA reagent | pRR64 E2 R251Q | This paper. |  | Derived through SDM of pRR64. Used to derive infectious virus through electroporation of RNA into cells. |
| recombinant DNA reagent | pRR64 E2 R251S | This paper. |  | Derived through SDM of pRR64. Used to derive infectious virus through electroporation of RNA into cells. |
| recombinant DNA reagent | pRR87 (Infectious cDNA clone of RRV DC5692) | PMID:21131014 |  | Used to derive infectious virus through electroporation of RNA into cells. |
| recombinant DNA reagent | pRR73 (Infectious cDNA clone of RRV T48-DC5692 E1/6K chimera) | PMID:21131014 |  | Used to derive infectious virus through electroporation of RNA into cells. |
| recombinant DNA reagent | pRR100 (Infectious cDNA clone of RRV T48-DC5692 E2/E3 chimera) | PMID:21131014 |  | Used to derive infectious virus through electroporation of RNA into cells. |
| recombinant DNA reagent | pONNV SG650 (Infectious cDNA clone of ONNV SG650) | PMID:9875334 |  | Used to derive infectious virus through electroporation of RNA into cells. |
| recombinant DNA reagent | pONNV SG650 E2 K200R (Infectious cDNA clone of mutant ONNV SG650) | This paper. |  | Derived through SDM of pONNV SG650. Used to derive infectious virus through electroporation of RNA into cells. |
| biological sample (*Ross River Virus*) | RRV SN11 | PMID:21430052 |  | Clinical isolate. Passed 1x on C6/36 cells and 1x on BHK-21 cells to generate out stock. |
| commercial assay or kit | QuikChange XL Site-Directed Mutagenesis Kit | Agilent | Cat#200517 |  |
| Genetic reagent (*M. musculus*) | μMT | Jackson Laboratory;  PMID:1901381 | Stock # 002288  RRID:IMSR_JAX:002288 |  |
| Genetic reagent (*M. musculus*) | C3^-/-^ | Jackson Laboratory;  PMID:8524789 | Stock # 029661  RRID:IMSR_JAX:029661 |  |
| Genetic reagent (*M. musculus*) | MARCO^-/-^ | PMID:15263032 |  | Dawn Bowdish |
| Genetic reagent (*M. musculus*) | SR-A1^-/-^ | Jackson Laboratory;  PMID:9069289 | Stock # 006096  RRID:IMSR_JAX:006096 |  |
| chemical compound, drug | PBS- and Clodronate-loaded liposomes | Liposoma Research | CP-005-005 |  |
| chemical compound, drug | Poly(I) potassium salt | Sigma | 26936-41-4 |  |
| chemical compound, drug | Poly(C) potassium salt | Sigma | 26936-40-3 |  |
| chemical compound, drug | Dextran | Sigma | 31392 |  |
| chemical compound, drug | Dextran Sulfate | Sigma | D6001 |  |
| commercial assay or kit | PureLink RNA mini kit | Life Technologies | CAT#12183025 |  |
| sequence-based reagent | CHIKV qPCR Primers | PMID:24131709 |  | FOR (5’-3’): TTTGCGTGCCACTCTGG  REV (5’-3’): CGGGTCACCACAAAGTACAA  TaqMan Probe (5’-3’): ACTTGCTTTGATCGCCTTGGTGAGA |
| sequence-based reagent | Sequence tagged (indicated with lowercase letters) RRV-specific RT primer | PMID:22972923 |  | 5′- ggcagtatcgtgaattcgatgcAACACTCCCGTCGACAACAGA-3′ |
| sequence-based reagent | RRV qPCR Primers | PMID:22972923 |  | RRV specific forward primer (5’-3’): CCGTGGCGGGTATTATCAAT  Tag sequence specific reverse primer (5’-3’): GGCAGTATCGTGAATTCGATGC  TaqMan Probe (5’-3’): ATTAAGAGTGTAGCCATCC |
| antibody | CHK-11 (Mouse monoclonal) | PMID: 23637602  Michael Diamond |  | Used in FFU assays at 500ng/ml |
| antibody | Secondary goat anti-mouse IgG-HRP  (Goat polyclonal) | Southern Biotech | Cat #1030-05  RRID:AB_2619742 | Used at 1:2000 in FFU assay |
| commercial assay or kit | TruBlue substrate | Fisher | Cat#50-78-02 |  |
| commercial assay or kit | RNAscope 2.5HD Assay-Brown | Advanced Cell Diagnostics | Cat# 322300 | Used for ISH of CHIKV RNA |
| other | V-CHIKV-sp-01 probe | Advanced Cell Diagnostics | Cat#481891 | CHIKV specific probe used for ISH of CHIKV RNA |
| other | Citrate pH 6.1 Target Retrieval Solution | Dako | Cat#S1699 | IHC |
| other | Dual Endogenous Enzyme Block | Dako | Cat#S2000389-2 | IHC |
| other | Protein Block | Dako | Cat#X090930 | IHC |
| antibody | F4/80 clone CI:A3-1  (Mouse monoclonal) | BioRad | Cat#MCA497   RRID:AB_2098196 | IHC; 1:100 |
| commercial assay or kit | VECTASTAIN Elite ABS reagent | Vector Laboratories | PK-6100  RRID:AB_2336819 | IHC |
| antibody | Biotinylated Anti-Rat IgG  (Goat polyclonal) | Vector Laboratories | BA-9401 | IHC; 1:100 |
| antibody | Anti-MHC-II (M5/114.15.2)  (Rat monoclonal) | Biolegend | Cat# 107605  RRID:AB_313320 | Flow Cytometry; 1:400 |
| antibody | Anti-Ly6C (HK1.4)  (Rat monoclonal) | Biolegend | Cat#128023  RRID:AB_10640119 | Flow Cytometry; 1:400 |
| antibody | Anti-F4/80 (BM8)  (Rat monoclonal) | Biolegend | Cat#123116  RRID:AB_893481 | Flow Cytometry; 1:400 |
| antibody | Anti-CD11b (M1/70)  (Rat monoclonal) | Biolegend | Cat#110224  RRID:AB_755986 | Flow Cytometry; 1:400 |
| antibody | anti-TCRβ (H57-597)  (Armenian Hamster monoclonal) | Biolegend | Cat#109228  RRID:AB_1575173 | Flow Cytometry; 1:200 |
| antibody | Anti-CD19 (6D5)  (Rat monoclonal) | Biolegend | Cat#115534  RRID:AB_2072925 | Flow Cytometry; 1:200 |
| antibody | Anti-CD11c (N418)  (Armenian Hamster monoclonal) | Biolegend | Cat#117337  RRID:AB_2562010 | Flow Cytometry; 1:400 |
| antibody | Anti-CD45 (30-F11)  (Rat monoclonal) | Biolegend | Cat#103149  RRID:AB_2564590 | Flow Cytometry; 1:400 |
| antibody | Anti-Ly6G (1A8)  (Rat monoclonal) | Biolegend | Cat#127624  RRID:AB_10640819 | Flow Cytometry; 1:400 |
| antibody | Anti-NK1.1 (PK136)  (Mouse monoclonal) | eBioscience | Cat#45-5941-82 | Flow Cytometry; 1:400 |
| Strain, strain background (*Aedes aegypti*) | Aedes aegypti mosquitoes | Collected in Poza Rica, Mexico F18-20  PMID:26658798 |  | Used in vector competence assays and competition assays |
| sequence-based reagent | 6106F and 6794R |  | PCR Primers (for competition assays) | 6106 FOR (5’-3’): atatctagacatggtgga  6794 REV (5’-3’): tatcaaaggaggctatgtc |
| sequence-based reagent | CHIKV-ApaI |  | PCR Primers (for SDM to make genetically marked CHIKV) | CHIKV-ApaI FOR (5’-3’): ctaaactaaaggggcccaaagcagcagcgctgt  CHIKV ApaI REV (5’-3’): acagcgctgctgctttgggcccctttagtttag |
| commercial assay or kit | Mag-Bind Viral DNA.RNA 96 kit | Omega Bio-Tek | Cat# M6256-02 | For RNA extractions from mosquito samples |
| other | KingFisher Flex Magnetic Particle Processor | Thermo Fisher Scientific |  | For RNA extractions from mosquito samples |
| software, algorithm | Gene Tools | Syngene |  | Used to quantify band intensities in competition experiments |
